# Quantification of Information Encoded by Gene Expression Levels During Lifespan Modulation Under Broad-range Dietary Restriction in *C. elegans*

**DOI:** 10.3791/56292

**Published:** 2017-08-16

**Authors:** Dhaval S Patel, Giovanni Diana, Eugeni V. Entchev, Mei Zhan, Hang Lu, QueeLim Ch'ng

**Affiliations:** ^1^Centre for Developmental Neurobiology, King's College London; ^2^Interdisciplinary Bioengineering Graduate Program, Georgia Institute of Technology; ^3^Wallace H. Coulter Department of Biomedical Engineering, Georgia Institute of Technology; ^4^School of Chemical & Biomolecular Engineering, Georgia Institute of Technology

**Keywords:** Genetics, Issue 126, Dietary Restriction, High-throughput Imaging, Information Theory, Neuroscience, Microfluidics, Neural Coding, Gene Expression

## Abstract

Sensory systems allow animals to detect, process, and respond to their environment. Food abundance is an environmental cue that has profound effects on animal physiology and behavior. Recently, we showed that modulation of longevity in the nematode *Caenorhabditis elegans* by food abundance is more complex than previously recognized. The responsiveness of the lifespan to changes in food level is determined by specific genes that act by controlling information processing within a neural circuit. Our framework combines genetic analysis, high-throughput quantitative imaging and information theory. Here, we describe how these techniques can be used to characterize any gene that has a physiological relevance to broad-range dietary restriction. Specifically, this workflow is designed to reveal how a gene of interest regulates lifespan under broad-range dietary restriction; then to establish how the expression of the gene varies with food level; and finally, to provide an unbiased quantification of the amount of information conveyed by gene expression about food abundance in the environment. When several genes are examined simultaneously under the context of a neural circuit, this workflow can uncover the coding strategy employed by the circuit.

**Figure:**
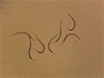


## Introduction

All organisms need to be able to sense and respond to changes to the environment to ensure their survival. In animals, the nervous system is the primary detector and transducer of information about the environment and coordinates the physiological response to any change that might affect the organism's survival^1^. Food abundance is an environmental cue that is well studied in multiple contexts that not only regulates food-related behaviors, such as foraging^2^, but also impacts the longevity of an animal. The modulation of lifespan by changes in food abundance is a phenomenon known as dietary restriction (DR), and has broad evolutionary conservation^3^.

The nematode *Caenorhabditis elegans* is a powerful model system for addressing fundamental biological questions. A plethora of techniques have been developed that allow for manipulation of the worm genome, such as RNAi and* in vivo* gene editing techniques. The small physical size of the worm and its optical transparency also lend themselves to* in vivo* imaging of both transcriptional and translational fluorescent reporters and utility of high-throughput technologies such as microfluidics^4^. Together, these tools can be harnessed to examine how neural circuits direct animal behavior.

*C. elegans* is a bacterivore and several methods have been published that allow for the precise control of food abundance by manipulating bacterial concentration^5^,^6^,^7^,^8^. Within the *C. elegans* research community, DR has been studied in two different contexts. The first can be termed 'classical DR', as it mirrors the changes seen in response to decreasing food levels in other organisms. In this context, decreasing food abundance from *ad libitum* levels results in an increasing lifespan up until an optimum is reached, after this point longevity decreases with further reduction of food^6^,^7^,^9^. The second context under which DR has been studied in *C. elegans* is dietary deprivation in which the longevity of the worms is increased by the complete removal of any bacterial food source^10^,^11^. In Entchev et al. (2015)^12^, we showed that the complexity in DR resulting from these two different paradigms can be examined simultaneously under a context we term 'broad-range DR'. By using the protocol outlined below, we identified a new class of genes involved in DR that bidirectionally modulate the lifespan response to food abundance and are involved in neural circuits that sense food^12^ (Figure 1).

The response of an animal to changes in the environment integrates a sequence of biological processes that link the sensory system to complex regulatory interactions conveying environmental information to physiology. Although the mechanistic details of such "information flow" are often unknown, genetic tools can be used to acquire an insight into how this complex computation is organized among different biological components. In our recent work, we showed that *daf-7* and *tph-1* are involved in the transmission of environmental information about food abundance through a food-sensing neural circuit that modulates lifespan in *C. elegans*^12^,^13^. By applying the mathematical framework of information theory^14^, we were able to quantify the amount of environmental information, in terms of bits, that is represented by the gene expression changes in *daf-7* and *tph-1* in specific neurons across different food levels. From this, we were then able to uncover the encoding strategy employed by this neural circuit and how it is genetically controlled (Figure 2).

In the following protocol, we outline the steps required to understand what the effects of genes of interest expressed in specific neurons are and how they participate to the flow of food information from environment to lifespan. Broadly, our framework is split in two experimental protocols and a computational workflow. For the experimental aspects, it is critical to have mutants of the genes of interest that can be examined under broad-range DR. Faithful transcriptional reporters are also necessary to quantify the expression level of the genes at different food levels. To be able to carry out the computational analysis discussed in our method, the dataset needs to be of sufficient size to provide meaningful estimates of expression distributions. Even though we provide template source codes for the analyses, the user needs to be familiar with the language of information theory that is extensively used throughout our computational framework. The source codes are written in R and C++. Therefore, a certain level of programming proficiency is also required to apply them in a meaningful way.

## Protocol

### 1. Preparation of Bacterial Cultures and Plates for General Worm Culture

1. Prepare 100 mL of lysogeny broth (LB) media in a 250 mL glass bottle and sterilize by autoclaving.
2. Inoculate the bottle with a single colony of the *Escherichia coli* strain OP50 and incubate at 37 °C overnight, and then store culture at 4 °C until needed.
3. Prepare 5 L of sterile nematode growth medium (NGM) agar^15^ and aliquot 12 mL volumes into sterile 6 cm plastic Petri dishes.
4. Allow the agar to set overnight and then store at 4 °C until needed.
5. Seed NGM plates at least 3 days prior to use with 225 μL of the refrigerated OP50 culture. Store the plates at 20 °C until needed.

### 2. Preparation of Bacterial Cultures and Dilutions for Experiments

1. Prepare two lots of 500 mL of LB media in a 2 L Erlenmeyer flask and sterilize by autoclaving.
2. Inoculate each flask with a single colony of OP50 and grow at 37 °C shaking at 200 rpm for around 14 h.
3. Supplement the cultures with the antibiotic streptomycin to a final concentration of 50 μg/mL. Continue shaking for 30 min at 37 °C then place the flasks on ice for 15 min.
4. Transfer 450 mL of each culture into separate sterile centrifuge bottles (ideally 500 mL capacity bottles to avoid splitting the cultures). Retain the leftover culture on ice, as it will be used to determine the concentration of the bacteria.
5. Spin down the bottles at 4500 x *g* in a refrigerated centrifuge set at 4 °C for 25 min. Discard supernatant and store the bottles on ice.
6. Dilute 100 μL of leftover culture from each flask in 900 μL of sterile LB. Use 1 mL of sterile LB to zero the spectrophotometer, and then determine the OD_600_ of the 10-fold dilution of each culture. If for example, the OD_600_ of the 10-fold dilution of the overnight culture is 0.28 then the culture had an actual OD_600_ of 2.8.
7. Use a working stock solution of bacteria of 1.12 x 10^10^ OP50 cells/mL, which equates to an OD_600 _of 56. Therefore, using the example OD_600_ of 2.8 from above, resuspend the pellet from the 450-mL culture in a 20^th^ of the original volume, which in this case is 22.50 mL. Resuspend bacteria with a sterile S basal solution supplemented with streptomycin to 50 μg/mL (SB + strep).
8. Resuspend the pellet in the appropriate volume of sterile SB + strep.
9. Make all subsequent concentrations used in experiments from serial dilutions of the working stock in SB + strep using the dilution factors listed in Table 1.

### 3. Setting Up Lifespan Experiments

NOTE: Lifespan assays are performed on 6 cm treated tissue culture filled with 12 mL of NGM agar supplemented with streptomycin and carbenicillin (NSC), both at a final concentration of 50 μg/mL. These tissue culture plates are well suited for lifespan assays at no or very low bacterial concentrations, as the worms are significantly less prone to sticking to the wall of the plate and desiccating. The use of two different antibiotics prevents the OP50 strain from developing drug-resistance, which is critical in controlling the bacterial concentration on the plate. Also, by inactivating bacteria with antibiotics, we minimize the effects on worm lifespan due to pathogenic infection^16^. This allows us to consider only the food-related components of bacterial concentration in these experiments. Many *C. elegans* ageing studies supplement NGM plates with the chemical fluoro-2′-deoxyuridine (FUdR) to render the adults sterile. However, the use of FUdR can be problematic as its use can cause several confounding issues with longevity assays^17^,^18^,^19^,^20^,^21^. Entchev et al. (2015)^12^ circumvented this issue by exposure of L4 larvae to RNAi of *egg-5*^22^,^23^, which inhibits the oocyte-to-embryo transition by blocking the formation of the eggshell of fertilized *C. elegans* oocytes resulting in their death.

1. Culture the *C. elegans* strains that will be assayed on seeded NGM plates at 20 °C. Allow the plates to starve out to ensure that all animals are grown in a uniform fashion and to potentially account for any transgenerational effects of developmental conditions.
2. Transfer starved L1 larvae to new seeded NGM plates by cutting out a small piece of agar (5 mm x 5 mm) from the starved plate using a sterile scalpel and placing it down on a new plate, a process referred to as 'chunking' by the *C. elegans* research community^15^. Grow the worms till they reach the L4 stage. Five L4 larvae per strain are then transferred to individual seeded NGM plates and kept at 20 °C. These animals represent the P0 generation.
3. Use L4 progeny of the P0 generation to set up the F1 generation. Individually place F1 L4 larvae of the wild type N2 strain on to 30 seeded NGM plates. NOTE: For mutant strains, the number of plates required depends on their growth rate. For example, *daf-7(ok3125)* animals undergo a transient dauer arrest at 20 °C. Thus, this strain produces less L4 larvae than a plate of N2 wild type worms.
4. To compensate for this difference, set up *daf-7(ok3125)* plates up a day earlier than N2 plates and at a greater number, a good working ratio is 3:1.
5. Transfer synchronized L4-stage progeny of the F1 parents to NGM plates supplemented with 1 mM IPTG and 50 µg/mL carbenicillin (RNAi plates) that were seeded with 225 µL of HT115 bacteria expressing dsRNA targeting the *egg-5* gene and kept at 20 °C for 24 h. At least 360 worms per strain, kept at a density of 15 animals per plate, are needed per experiment.
6. After the *egg-5 *RNAi treatment, transfer the worms to NSC plates seeded with 225 µL of the streptomycin-treated OP50 at a concentration of 2 x 10^9^ cells/mL (**Table 1**) for an additional 24 hours at 20 °C. To avoid physical damage to the worms during this and all subsequent transfers, gently scoop animals up from underneath the worm using a very thin gently curved worm pick. Float the animals off the worm pick by immersing it into a 10 µL droplet of SB + strep placed on the surface of the new plate.
7. The next day, redistribute the worms to NSC plates representing each of the food levels listed in Table 1 as well as a set of NSC plates that contain no bacteria. Seed all NSC plates with 225 µL of the relevant concentration of bacteria, and seed the bacteria-free NSC plates with 225 µL SB + strep. Maintain the worms at a density of 15 animals per plate, resulting in at least four plates per food condition per strain. Shift the plates to the desired experimental temperature.
8. Transfer worms to fresh NSC plates seeded with appropriate food concentration according to the schedule outlined in **Table 2**.
9. Score animals for movement by gently prodding with a wire pick. Failure to respond is scored as death. Score animals for death at every transfer point and then daily after the last transfer point.

### 4. Setting Up Imaging Experiments

NOTE: The steps outlined in this section are sufficient to generate enough worms per strain for imaging one experimental food condition at a given temperature. This protocol can be scaled to fit the number of conditions that will be imaged on any given day. However, care should be taken in the experimental design to ensure that the timeframe is reasonable and that animals across different strains do not have an age difference greater than 12 hours on the day of imaging. It is highly recommended that the transcriptional reporters being imaged are single-copy transgenics, as this will more closely resemble the native regulation of the gene. The protocol outlined below, and summarized in **Table 3**, also provides a streamlined method for obtaining large synchronous age matched populations of strains, which can be used for other experimental workflows.

1. Culture animals in imaging experiments on 10 cm treated tissue culture plates to allow for a greater density of worms (∼100 animals per plate) than lifespan assays.
2. Fill the 10 cm plates with 30 mL of NGM agar plates and seed with five 225 µL aliquots of refrigerated OP50 culture in a cross-like formation.

### 5. Initial Culture of Reporter Strains

1. Culture the *C. elegans* strains that will be assayed on seeded NGM plates at 20 °C. Allow the plates to starve out to ensure that all animals are grown in a uniform fashion and to potentially account for any transgenerational effects of developmental conditions.
2. Chunk the starved L1 larvae to seeded NGM plates and grow till they reach the L4 stage. Transfer three L4 larvae per strain to two seeded NGM plates at 20 °C. These animals represent the P0 generation.
3. Use the L4 progeny of the P0 generation to set up the F1 generation. Place F1 L4 larvae of the wild type reporter strain on to 4 seeded NGM plates. For mutant strains, the number of plates required depends on their growth rate. Using the previous example of *daf-7(ok3125)*, reporter strains in this background require three times the number plates and need be set up two days before the wild type reporter strains, to account for differences in growth rate.

### 6. Egg-collection and Synchronization of Reporter Strains

NOTE: Some genes of interest in the *C. elegans* aging research community result in growth and egg-laying defects when mutated, which makes it more difficult to generate large synchronous populations of animals of different genotypes. For example, The *daf-7(ok3125)* mutation causes a severe egg-laying defect in comparison to the wild type N2 strain. Therefore, to get sufficient numbers of synchronous L4 larvae of different strains for quantitative imaging experiments requires a more robust methodology than manually picking worms. For this reason, transcriptional reporter strains were subjected to a sodium hypochlorite (NaClO)/ sodium hydroxide (NaOH) solution treatment of gravid adults to break open the animals and liberate their eggs, a process commonly referred to as 'bleaching' by the *C. elegans* research community^15^.

1. Account for differences in growth rates between strains and calculate when plates of each strain should be harvested and bleached. For an example of when wild type and *daf-7(ok3125)* reporter strains are bleached see **Table 3**.
2. Serially wash the worms of a given strain off the plates, on the appropriate day, using 15 mL of SB and collect in a sterile 15 mL tube. Allow the animals to naturally sediment and then adjust the volume of liquid to 7 mL.
3. Add 2 mL of 5% NaClO and 1 mL of 5 M NaOH to the tube containing the gravid adults. Gently rock the mixture at room temperature for no more than 3 min. The NaClO will kill the bacteria and worms, while the NaOH causes the worms to break apart releasing any fertilized eggs they contain into the liquid. The chitin eggshell around the embryos protects them from the effects of the treatment, as long as the exposure time remains relatively short. After the 3-min incubation, vortex the tube for ~30 s to facilitate further breakup of the worm carcasses.
4. After the 3-min incubation spin down the mixture at 1,000 x *g* for 1 min to pellet the eggs. Aspirate away most of the supernatant using a sterile glass pipette connected to a vacuum flask, leaving ~0.5 mL in each tube, so as not to disturb the eggs.
5. Resuspend the pellet with 9.5 mL of SB and then repeat the centrifugation step and resuspension steps an additional two times so that the eggs have been washed with SB a total of three times.
6. **After the final wash, pellet the eggs through centrifugation and then discard all but 0.5 mL of the supernatant. Resuspend the eggs in the remaining 0.5 mL of SB and then aliquot 100 µL on to three 10 cm seeded NGM plates. Distribute the 100 µL equally over all five bacterial lawns on each plate. For wild type strains, eggs should not be deposited at densities greater than 200 per plate.**
  1. Keep plates at 20 °C for 48 h and to obtain a highly homogenous population of L4 larvae. For strains with delayed growth phenotypes, it is advisable to deposit as many eggs as available and increase the incubation time. For example, keep* daf-7(ok3125)*-containing reporter strains at 20 °C for ~64 h to allow the eggs to hatch and reach the L4 stage.

### 7. Treatment of Reporter Strains with *egg-5* RNAi

1. Serially wash the worms off the three plates using 15 mL of SB and collect the liquid in a sterile 15 ml tube. Allow the L4 larvae to naturally sediment, and then aspirate all but ~0.5 mL of the liquid. In the wild type reporter strains, this step removes any larvae younger than L4. In mutant backgrounds, this step aids the removal of any arrested larvae such as dauers in the case of *daf-7(ok3125)*.
2. Resuspend the worms with 9 mL of SB. Again, monitor the sedimentation rate of the L4 larvae and then aspirate all but ~0.5 mL of the supernatant once most of the L4 larvae have pelleted. Repeat this process once more. Then aspirate all but ~0.5 mL of the liquid once the majority of L4 larvae have settled.
3. **Add 10 µL of sterile S basal supplemented with 0.1% Pluronic F-127 (SB + Plu) to the liquid containing the L4 larvae. This acts as a surfactant and prevents the larvae from sticking to the interior surface of plastic pipette tips.**
  1. Gently resuspend the larvae using a P200 low retention pipette tip and then aliquot 150 µL onto three 10 cm RNAi plates that are seeded with 5 x 225 µL of *egg-5* RNAi bacteria. Ensure that the worms are equally distributed across all five bacterial lawns.
4. Once the liquid has absorbed into the agar, remove any non-L4 larvae from the plates that were not eliminated by the washing procedure by manually picking them off. Then store the plates at 20 °C for 24 h.

### 8. Initiation of Broad-range DR

NOTE: After the 24 h *egg-5* RNAi treatment, the L4 larvae that were originally deposited on the plates will have become 1-day old adults.

1. Pick off any young larvae that escaped the prior manual removal step off at this stage, leaving only the 1-day old adults on the plates.
2. For each strain, wash the 1 day old adults from the three plates with 15 mL of sterile SB + strep into a 15 mL tube. Allow the worms to naturally sediment, and then aspirate all but 0.5 mL of the supernatant. Resuspend the worms with 9.5 mL of SB + strep and repeat the sedimentation and wash steps.
3. **After the final wash, allow the worms to sediment and then aspirate all but 0.5 mL of supernatant. Add 10 µL SB + Plu and gently resuspend the larvae using a P200 pipette tip and then aliquot 100 µL onto an NSC plate seeded with 5 x 225 µL bacteria at a concentration 2 x 10^9^ cells/mL.**
  1. Distribute the 100 µL evenly across all five bacterial lawns. Under a microscope estimate the number of animals present on the plate: the aim is to have between 100 - 150 worms on the plate.
  2. Determine the volume of liquid required to achieve a worm density within this range and then aliquot onto two additional plates. Adjust the number of worms on the first plate to also fall into this range and then store the plates at 20 °C for 24 h.
4. The next day, collect the 2 day old adults and distribute to new NSC plates seeded with the desired experimental concentration of food (**Table 1**), reusing the methods outlined in steps 2 and 3. Once the liquid is absorbed into the agar, shift the plates to the desired experimental temperature for 24 h.
5. The next day, collect the 3 day old adults and distribute to fresh NSC plates seeded with the same experimental concentration of food, reusing the methods outlined in steps 8.3 and 8.4. Once the liquid is absorbed into the agar, return the plates to the experimental temperature for 48 h.
6. Collect the 5 day old adults and distribute to fresh NSC plates seeded with the same experimental concentration of food, reusing the methods outlined in steps 8.3 and 8.4. Once the liquid is absorbed into the agar, return the plates to the experimental temperature for 24 h.

### 9. Microfluidic Imaging of Reporter Strains

1. **On day 6 of adulthood, image animals using a custom microfluidic platform^24^,^25^. Physically pick off animals from the three plates and suspend in a 5 mL cryogenic tube containing 4.5 mL of SB + strep.**
  1. Once the worms sediment, aspirate all but ~0.5 mL of the supernatant and resuspend the animals in 4 mL of SB + strep. This wash removes excess bacteria that might otherwise interfere with imaging. The worms are introduced into a custom microfluidic device via pressure driven flow^24^,^25^. Within the device, individual worms are directed into and trapped within an imaging channel gated by pressure-driven on-chip valves^26^ under the control of custom software.
2. Once a worm is trapped headfirst in the imaging channel, collect a fluorescent z-stack, with 50 sections in 2 µm steps, using a standard epifluorescence microscope with a 40X oil objective (1.3 NA) and a camera. Collect red and green fluorescent images for each of transcriptional reporters simultaneously using an emission splitter and stored for analysis. Image acquisition is automated using custom software.
3. Process the image automatically using custom MATLAB scripts^27^ (available at https://github.com/meizhan/SVMelegans). The Z-stacks are loaded into MATLAB and analyzed for to identify neuron-pairs and their locations within the imaging plane. The maximum projections are then computed, and a thresholding algorithm is utilized to locate individual fluorescent cells. Cell identification is then computed based on relative distances and locations within the worm's head.
4. To quantify reporter fluorescence, extract the three-dimensional volume around each cellular location from the z-stack. Integrate the intensity over a consistent number of the brightest pixels, which fully encapsulate the entire cell in all cases.
5. To eliminate interference from experimental condition-specific or strain-specific changes in the gut auto-fluorescence of each animal, calculate the background intensity for the cell pairs nearest the gut (ADF and ASI) via estimation of the mode of the intensity distribution in a volume around the neuron. Subtract this background intensity value from the integrated fluorescence to obtain the final output.

### 10. Data Assembly

NOTE: The fluorescence intensities of all neurons analyzed by the image processing software are combined into the filtered expression data file (FED) which is used to estimate the distribution profiles of gene expression (template R and C++ scripts are available at https://github.com/giovannidiana/templates).

1. Manually check each processed image to confirm correct identification for all cells. Images with erroneous cell identification must be recorded in an exclusion file. In Diana et al. (2017)^13^ the exclusion file consists of a binary table with '0' for correct and '1' for incorrect cell identification. The number of rows in the table is equal to the number of worms imaged with a column for each cell imaged (i.e ASI, ADF and NSM).
2. **Generate the FED file:**
  1. Run the bash script "gen_data+time" to generate cell-specific files combining the expression values obtained from each worm imaged filtered using the exclusion file. For each folder generated by the image processing software, the bash script reads the annotation file <folderprefix>_EXP.txt to extract the experimental conditions and the exclusion file <folderprefix>_X.csv to select only correctly identified neurons. Expression values are read from the files <folderprefix>_data_<neuron>.csv.
  2. Concatenate all files into "FED_split.dat" and sort it by the experiment batch code, worm label and cell identity.
  3. Run "sort" (C++ program, usage: ./sort FED_split.dat FED_merged.dat) to select entries with non-zero fluorescence for each cell and combine them into single rows in FED_merged.dat.

### 11. Estimation of Information Encoded

NOTE: The following procedure describes how to quantify the information about specific environmental conditions encoded by the set of gene expressions. In Diana *et al. *(2017)^13^, the information encoded about food abundance in the environment was examined, however, the method itself is applicable to any discrete number of environmental states. The essential ingredient to quantify information theoretic variables such as information entropies or redundancy is the joint probability distribution of the neural responses under the set environmental stimuli considered. To perform such estimation, it is crucial to have a sufficient sampling of the response across populations of worms. Gaussian distributions can be estimated from relatively small samples; however, it is important to have an idea of the expected shape of the expression distribution to quantify the appropriate sample size for a reliable density estimation. Due to the unavoidable variability across different trials, it is essential to check that the central values of the distributions obtained from different repeats of the same experiment are not systematically shifted or that any of the statistical features of the expression distribution are not significantly altered across trials. In case the trial-to-trial variability is compatible with the variability within each trial, it is crucial to balance the number of trials versus the number of worms within trials to average out those environmental/biological factors that affect trial-to-trial variability. Undersampling those factors could heavily bias the information-theoretic analysis.

1. As the R script "code3D.R" provides a template for generating three-dimensional densities based on the file "FED_merged.dat", modify this template according to the specific FED header format. The list "HeaderNames" represents the names of each field in the FED file while "RONames" is the list of the readouts. The script uses the R package 'ks'^28^,^29^to estimate multivariate distributions within a hypercubic grid with GS bins in each dimension subdividing the range between minimum and maximum values in the dataset for each readout. When the internal variable "group" is set to 0 all data are used for density estimation. When the "group" label is between 1 to 5 the dataset is divided into 5 disjoint sets, and the expression densities are estimated from the 80% of the data resulting from the exclusion of one of the five sets. This feature is used later on to estimate uncertainties. NOTE: Usage of code3D.R: Rscript code3D.R <GT> <food> <GS> <outfolder> <label> <group> <frac> where GT represents the genotype, food is the environmental condition, outfolder is the pre-existing folder where the distributions will be stored, label is a filename prefix and frac is the fraction of the dataset used.
2. For each environmental condition generate the multivariate distributions with different grid sizes GS (*e.g. *20,30,40 bins). To reduce computational load, smaller values of GS are preferable when finer resolutions do not change significantly the information estimation. Gene-expression distribution are written in the folder <outfolder> specified when running code3D.R as single column text files with filename structure <label>_<GT>_<food>_GS<GS>_group<group>.dat. NOTE: The previous steps provide the estimate of the conditional probability distributions 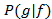 where g denotes the vector of all read-outs and f is the environmental condition. However, to calculate the mutual information between gene expression and the environment^30^,^31^. 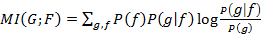 We need the distribution of the input 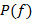 which also determines the (input-) averaged gene-expression 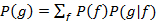. When the input distribution is not directly available, a meaningful quantity to characterize the coding features is the channel capacity, which can be obtained by maximizing the mutual information across all possible input distributions.
3. Using the gene-expression distribution, apply the Arimoto-Blahut^32^ algorithm to estimate the channel capacity of the system and the distribution of the environmental input that maximizes information. An example of implementation of the algorithm can be found in the C++ program "Ccap3D.cpp" for the three-dimensional gene-expression code analyzed in Diana et al. (2017)^13^. Example: if distributions from the genotype GT123 are obtained from the 80% of the data (group 3) with grid size equal 30 and stored in the folder "./pdf/" using a prefix label="PDF". The command to calculate the information encoded in the GT123 background is ./Ccap3D GT123 pdf PDF 30 3 NOTE: The estimation of the information encoded by the system is affected by multiple sources of uncertainty, including the choice of the density estimation algorithm and sample size bias. Steps 11.1-11.2 should be repeated using different density estimation methods to evaluate the size of systematic bias introduced by each algorithm.
4. To estimate the uncertainty due to sample size, recalculate information from steps 11.1-11.2 with groups between 1 to 5. The variability across these five independent samplings of the 80% of the data will reflect the impact of sample size on information estimation.
5. Calculate information using steps 11.1-11.2 over increasing fraction of the data (as in step 5) to correct for sample-size bias (Jack-knife method)^33^.

### 12. Calculation of Redundancy, Noise and Signal Correlation

1. Use the optimal input distributions obtained from the estimate of the maximal information to calculate the mutual information 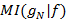 between input and gene expression response of each neuron *N*. The C++ source file "GetMI1D.c" is a sample program to obtain marginal mutual information from the joint probability distributions.
2. Calculate redundancy by taking the sum of the mutual information for each neuron obtained above and then subtract the channel capacity.
3. Calculate the "shuffle" information term 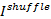
13,34. A template C++ source file can be found in "GetShuffle.c".
4. Use the "shuffle" term to calculate signal and noise correlation. For the signal correlation we have 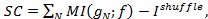 and for the noise correlation we have 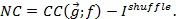.
5. Uncertainties on redundancy, obtain noise and signal correlation as for the total information (step 11.3 of the previous section) from multiple samplings of the 80% of the data and taking the standard deviation.

## Representative Results

By conducting lifespan experiments on the mutants of the genes of interest alongside the wild type N2 strain, one can establish whether these genes have a role in the food response to broad-range DR. The wild type response should be comparable to the one depicted in Figure 1**A**. Any modulation of this response by the mutants, reflected by a non-uniform effect across food conditions, indicates that these genes affect the ability of the worm to correctly respond to changes in food abundance, at which point further investigation of the expression responses of these genes to broad-range DR is warranted. If, however, the longevity response of the mutants is not significantly different from the wild type then the genes have no role in transducing the effects of broad-range DR, at least at the level of mean lifespan. If the mutations cause a uniform shift of the whole lifespan response then the genes have a food-independent effect on longevity. This does not rule out the possibility that the expression of the genes of interest is food-responsive, in which case the information carried by these genes is not transmitted to lifespan.

The next stage of the protocol is to determine how expression levels change under broad-range DR for the genes of interest. In Figure 1**B**, we illustrate this through the expression levels of a transcriptional reporter of *daf-7,* which shows a response to changes in food level in the ASI sensory neurons. In a *daf-7(-)* mutant, the expression response of the transcriptional reporter is altered. If the genes of interest are truly food-responsive at the level of lifespan then one can expect that their expression will also change with food. Correspondingly, a transcriptional reporter in the mutant background should have an altered expression profile in response to broad-range DR. However, it is also possible that the transcriptional reporter of the gene of interest in a wild type background does not show any food-responsive changes in expression. In this situation, this may indicate a post-transcriptional regulatory effect that falls outside the scope of this protocol.

In Diana et al. (2017)^13^, we extracted expression values for *daf-7* in ASI and *tph-1* in ADF and NSM. In Figure 2**A**, we illustrate the estimation of the expression distribution in ASI and ADF for a given food level. Having multiple readouts from single worm images allows us to analyze not only the amount of information encoded independently by each neuron but also the combinatorial information of the whole neural circuit (Figure 2**B-2C**). Combining these two information-theoretic measures allows us to characterize the system in terms of the encoding strategy employed by the neurons to convey information about food. The amount of redundancy in the circuit can be obtained by taking the sum of the mutual information for each neuron and subtracting the joint mutual information (channel capacity) obtained by considering the combinatorial readouts of the circuit. A positive value of such difference denotes a redundant character of the encoding strategy because the cumulative information among the parts is larger than the actual information encoded by the whole circuit. Conversely a negative value reflects a synergistic strategy because the true information encoded is larger than the sum of its components (Figure 2**B**). Information and redundancy can be compared across different genotypes to explore possible higher order roles of gene regulation, for instance in Diana *et al. *(2017)^13^ the effect of *daf-7* mutation switches the encoding strategy from redundant to synergistic (Figure 2**C-2D**).

**Figure:**
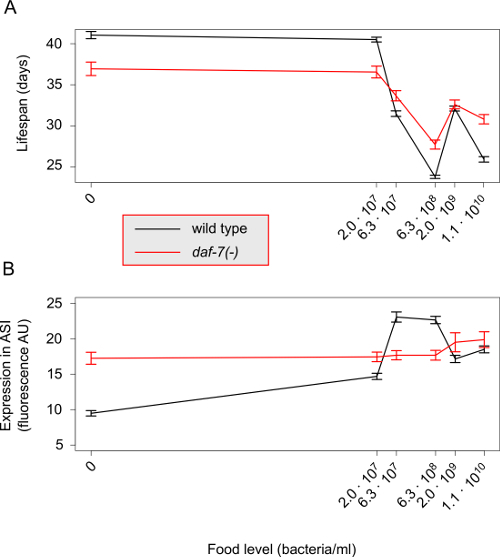

Figure 1**: Response of lifespan and gene expression under broad-range DR. **(**A**) The mean lifespan of the wild type N2 strain (black line) displays a complex response to broad-range DR. The magnitude of this response is attenuated in a null mutant of the *daf-7* gene (red line). Error bars represent standard error of the mean, pooled data from Entchev *et al. *(2015)^12^. (**B**) The mean expression levels of a transcriptional reporter for the *daf-7* gene in wild type background (black line) also display a complex non-monotonic response to broad-range DR. In *daf-7(-)* genetic background the expression of this transcriptional reporter is highly attenuated and shows little response to changes in food level. Error bars represent standard error of the mean, data from a single trial in Entchev *et al.* (2015)^12^. Please click here to view a larger version of this figure.

**Figure:**
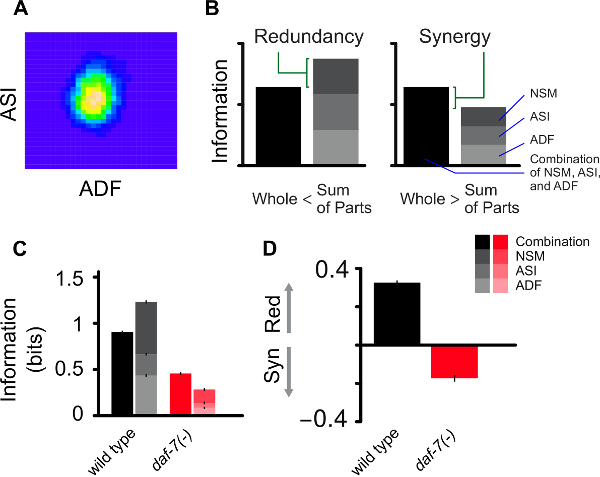

Figure 2**:Computational methodology. **(**A**) Illustration of two-dimensional density estimation of *tph-1* expression in ADF and *daf-7* expression in ASI as obtained from the 'ks' R package using a grid dimension 30 x 30. (**B**) Visualization of the information encoded by the joint expression of* tph-1* and *daf-7* (whole) and individually (sum of parts) for ADF, ASI and NSM neurons. Redundant and synergistic characters of the encoding are represented by the difference between the height of the stacked bars on the right and the information encoded by the full circuit. (**C**) Comparison between food information encoded by wild type animals and *daf-7(-) *mutants. (**D**) The reduction of mutual information observed in the mutants is accompanied by a switch towards synergistic encoding. Panels B-D are adapted from Diana *et al. *(2017)^13^. Please click here to view a larger version of this figure.

**Table:** 

**Bacterial Concentration (cells/ml)**	**Optical Density (600nm)**	**Dilution Factor (From Previous)**
1.12E+10	56.000	0.00
2.00E+09	10.000	5.60
6.32E+08	3.160	3.16
6.32E+07	0.316	10.00
2.00E+07	0.100	3.16
0 (S basal)	0.000	NA

**Table 1: Food levels and dilution factors used in broad-range DR. **Bacterial concentrations (cells/mL) used in the broad-range DR protocol, along with their respective OD_600_ measurements and the dilution factor required to achieve each concentration from the previous one.

**Table:** 

	**Experimental Temperature of Lifespan**
**Day**	**12.5°C**	**15°C**	**17.5°C**	**20°C**	**22.5°C**	**25°C**	**27.5°C**
**-12**	Chunk all strains to fresh NGM plates and maintain at 20°C
**-11**							
**-10**	Set up P0 generation of daf-7(-) strains and maintain at 20°C (1 L4 per plate, 5 plates)
**-9**	Set up P0 generation of wild type strains and maintain at 20°C (1 L4 per plate, 5 plates)
**-8**							
**-7**							
**-6**							
**-5**	Set up F1 generation of daf-7(-) strains and maintain at 20°C (1 L4 per plate, 90 plates)
**-4**	Set up F1 generation of wild type strains and maintain at 20°C (1 L4 per plate, 30 plates)
**-3**							
**-2**							
**-1**							
**0**	Pick F2 L4 larvae to >egg-5 RNAi plates and maintain at 20°C (15 L4 per plate, 24 plates)
**1**	Move 1-day old adults to NSC plates seeded with 2.0E+9 cells/ml food level and maintain at 20°C
**2**	Move 2-day old adults to NSC plates seeded with experimental food conditions and to experimental temperature
**3**	Transfer	Transfer	Transfer	Transfer	Transfer	Transfer	Transfer
**4**							
**5**	Transfer	Transfer	Transfer	Transfer	Transfer	Transfer	Transfer
**6**							
**7**	Transfer	Transfer	Transfer	Transfer	Transfer	Transfer	Transfer
**8**							
**9**	Transfer	Transfer	Transfer	Transfer	Transfer	Transfer	Transfer
**10**							
**11**	Transfer	Transfer	Transfer	Transfer	Transfer	Transfer	
**12**							
**13**							
**14**	Transfer	Transfer	Transfer	Transfer			
**15**							
**16**							
**17**							
**18**	Transfer	Transfer					
**19**							
**20**							
**21**							
**22**	Transfer						

**Table 2: Schedule for lifespans conducted at different temperatures. **Schematic outline of the steps needed to set up broad-range DR lifespan experiments at different temperatures using *daf-7(-)* and wild type strains as examples. The number of transfers to fresh plates of each experimental food level decreases with increasing temperature. This is to account for the fact that animals at higher temperatures are aging more rapidly and so a more prone to physical damage per transfer.

**Table:** 

**Days**	**Imaging Pipeline**
**-14**	Chunk reporter strains in daf(-) background. Maintain at 20 °C.
**-13**	Chunk reporter strains in wild type background. Maintain at 20 °C.
**-12**	Set up P0 generation of daf-7(-) reporter strains. Use 3 L4 larvae per 10cm NGM plate. Use 2 plates and maintain at 20 °C.
**-11**	
**-10**	Set up P0 generation of wild type reporter strains. Use 3 L4 larvae per 10cm NGM plate. Use 2 plates and maintain at 20 °C.
**-9**	
**-8**	Set up F1 generation of daf-7(-) reporter strains. Use 3 L4 larvae per 10cm NGM plate. Use 12 plates and maintain at 20 °C.
**-7**	
**-6**	Set up F1 generation of wild type reporter strains. Use 3 L4 larvae per 10cm NGM plate. Use 4 plates and maintain at 20 °C.
**-5**	
**-4**	
**-3**	Bleach daf-7(-) reporter strains in afternoon (~5pm) and deposit eggs on 3 10cm NGM plates and maintain at 20 °C.
**-2**	Bleach wild type reporter strains in morning (~10am) and deposit eggs on 3 10cm NGM plates and maintain at 20 °C.
**-1**	
**0**	Wash L4 to 10 cm egg-5 RNAi plates. Use 3 plates per strain and maintain at 20 °C.
**1**	Wash 1-day adults to NSC plates seeded with 2.0E+9 cells/ml. Use 3 plates per strain and maintain at 20 °C.
**2**	Wash 2 day adults to NSC plates seeded with experimental food levels. Use 3 plates per strain and shift to experimental temperature.
**3**	Transfer to fresh NSC plates. Use 3 plates per strain and maintain at experimental temperature.
**4**	
**5**	Transfer to fresh NSC plates. Use 3 plates per strain and maintain at experimental temperature.
**6**	Pick animals off plates and prepare for imaging.

**Table 3: Schedule for imaging pipeline. **Schematic outline of the steps needed to set up broad-range DR imaging experiments using fluorescent transcriptional reporter strains in *daf-7(-)* and wild type backgrounds at different temperatures as examples.

## Discussion

Here, we present a new method for dietary restriction that encapsulates a much broader range of food concentrations than previously published protocols. This method links two previously separate phenomena seen in *C. elegans* DR literature, bacterial deprivation and classical dietary restriction, allowing both dietary effects to be studied under one protocol. Using the new broad-range DR paradigm, we present a general framework for examining single cell gene expression in response to a specific environmental cue and determining how this cell encodes information. Our framework consists of two experimental protocols that illustrate how to perform lifespans and quantitative imaging, respectively, under broad-range DR. Data from these experimental protocols can then be examined with the computational analyses provided in this framework to quantify the information encoded by changes in the gene expression levels or lifespans across different food conditions.

Lifespan experiments using broad-range DR paradigm involve six distinct food levels (**Table 1**). This necessitates a more labor-intensive approach than examining longevity under fewer food levels, such as dietary deprivation^10^,^11^ or using the *eat-2* genetic background^35^. However, examining at lifespan under a single condition can limit the interpretations of a gene's role in DR. For example, we recently showed that *daf-7* mutants have a bidirectional attenuation of the response to food concentration compared to wild type animals^12^ (**Figure 1A**). In the absence of food, *daf-7* mutants display a shortening of their lifespan compared to wild type animals. If we had only considered dietary deprivation, we would have interpreted that the *daf-7* gene as being necessary for only lifespan extension*,* when in fact *daf-7* role is more complex. Therefore, the critical outcome of this part of the protocol is to establish whether a gene of interest is involved in modulating the overall response of lifespan to changes in food abundance.

One major advantage of this protocol compared to other methods is that it uses a novel method to eliminate progeny production in the animals undergoing lifespan analysis. Most studies use the drug FuDR to inhibit proliferation of the germline in adults rendering them sterile. However, recent studies have shown FuDR treatment can have condition- and gene-specific effects on lifespan^17^,^18^,^19^,^20^,^21^, calling into question its general applicability. In this protocol, elimination of progeny production is achieved through a 24 h treatment of animals with RNAi targeting the *egg-5* gene, which inhibits the formation of the chitin eggshell of fertilized *C. elegans* oocytes resulting in their death^22^,^23^. The advantage of this method is that it is very late-acting and so does not interfere with the germline, which is a major regulator of longevity in *C. elegans*.

One potential caveat of the broad-range DR protocol is its reliance on the use of the antibiotics to control bacterial proliferation to ensure tight control of bacterial concentration. Bacterial proliferation within the gut of the worm is known to be a major cause of death in *C. elegans*^16^. Thus, the use of bacteriostatic antibiotics, such as carbenicillin, in NGM agar prevents bacterial proliferation and increases lifespan of worms compared to non-antibiotic controls^16^. Certain types of antibiotics, such as rifampicin^36^ and members of the tetracycline family^37^,^38^, have been shown to extend lifespan in *C. elegans* independently of their effect on bacterial proliferation. However, there is no evidence in the literature that either carbenicillin or streptomycin can increase longevity independently of their effect on bacterial proliferation.

Lifespan can be viewed as the output of a complex computation where environmental information, routed by gene-expression in neuronal networks, is transmitted to physiology. Our protocol provides a methodology to understand how specific genes affect this flow of environmental information. To address this question, we need reliable image processing to determine the distribution of gene expression responses at the single-cell level. Being able to estimate not only the average response of gene expression to changes in food abundance but also the full statistical distribution from large populations represents an important requirement for the applicability of our method. This accurate description of gene expression responses to food abundance allows the application of information theory to quantify the information encoded by the specific neurons as well as the coding strategy employed by the neural circuit.

The imaging and computational aspects of the methods outlined in this protocol are applicable to a greater set of biological contexts. In our work, we focused on a small neural network involved in food sensing, however, the analyses of information-processing features are not limited to a specific cell type or specific environmental cues. In the future, these methodologies can potentially be extended to a larger variety of input variables, affecting any physiological output. These approaches will contribute to a greater understanding of how gene regulatory networks encode, process and transmit information.

## Disclosures

The authors have nothing to disclose.
